# Mesenchymal chondrosarcoma: An Australian multi‐centre cohort study

**DOI:** 10.1002/cam4.4849

**Published:** 2022-05-23

**Authors:** Madeleine C. Strach, Peter S. Grimison, Angela Hong, Richard Boyle, Paul Stalley, Rooshdiya Karim, Elizabeth A. Connolly, Susie Bae, Jayesh Desai, Philip Crowe, Nimit Singhal, Vivek A. Bhadri

**Affiliations:** ^1^ Chris O'Brien Lifehouse Sydney New South Wales Australia; ^2^ Royal Prince Alfred Hospital Sydney New South Wales Australia; ^3^ The University of Sydney Faculty of Medicine and Health Sydney New South Wales Australia; ^4^ Peter MacCallum Cancer Centre Melbourne Victoria Australia; ^5^ Sir Peter MacCallum Department of Oncology The University of Melbourne Melbourne Victoria Australia; ^6^ Prince of Wales Hospital Sydney New South Wales Australia; ^7^ Cancer Centre, Royal Adelaide Hospital and Department of Medicine University of Adelaide Adelaide South Australia Australia; ^8^ Present address: The Christie NHS Foundation Trust Manchester UK

**Keywords:** chemotherapy, mesenchymal chondrosarcoma, prognostic factors, treatment outcome

## Abstract

**Background:**

Mesenchymal chondrosarcoma (MCS) is an ultra‐rare sarcoma that follows a more aggressive course than conventional chondrosarcoma. This study evaluates prognostic factors, treatments (surgery, chemotherapy, and radiation), and outcomes in an Australian setting.

**Methods:**

We collected demographics, clinicopathological variables, treatment characteristics, and survival status from patients with MCS registered on the national ACCORD sarcoma database. Outcomes include overall survival (OS) and progression‐free survival (PFS).

**Results:**

We identified 22 patients with MCS between 2001–2022. Median age was 28 (range 10–59) years, 19 (86%) had localised disease at diagnosis of whom 16 had surgery (84%), 11 received radiation (58%), and 10 chemotherapy (53%). Ten (52%) developed recurrence and/or metastases on follow‐up and three patients with initial metastatic disease received surgery, radiation, and chemotherapy. At a median follow‐up of 50.9  (range 0.4–210) months nine patients had died. The median OS was 104.1 months (95% CI 25.8–182.3). There was improved OS for patients with localised disease who had surgical resection of the primary (*p* = 0.003) and those with ECOG 0–1 compared to 2–3 (*p* = 0.023) on univariate analysis.

**Conclusions:**

This study demonstrates contemporary Australian treatment patterns of MCS. The role of chemotherapy for localised disease remains uncertain. Understanding treatment patterns and outcomes help support treatment decisions and design of trials for novel therapeutic strategies.

## Graphical abstract

This study demonstrates current treatment patterns and clinical outcomes for mesenchymal chondrosarcoma, an ultra‐rare tumour. This helps support treatment decisions and design of trials for novel therapeutic strategies.

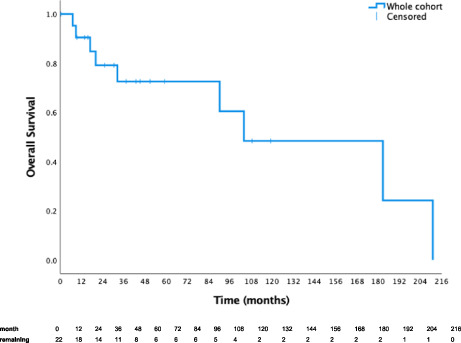

## 1 INTRODUCTION

Chondrosarcomas account for 20% of primary bone tumours with a global incidence of one in 200,000.^1^ Conventional chondrosarcoma is the most common subtype, with non‐conventional subtypes including mesenchymal, myxoid and dedifferentiated accounting for 2%–10%.^2^, ^3^ Mesenchymal chondrosarcoma (MCS) is an ultra‐rare sarcoma that typically follows a more aggressive course than conventional chondrosarcomas, with only 226 cases of MCS reported in the Surveillance, Epidemiology, and End Results (SEER) database between 1973 and 2013.^3^, ^4^

MCS morphologically appears as a small round cell sarcoma with islands of chondroid matrix and populations of dedifferentiated spindle cells.^5^ MCS diagnosis can be confirmed by the presence of NKX3.1 on immunostaining^6^ and detection of the *NCOA2* gene rearrangements and fusions (such as *HEY1‐NCOA2*) and can now be incorporated into the group of translocation‐related sarcomas,^7^ which may yield biological insight into future treatments.^8^, ^9^

MCS occurs slightly more in young males.^3^, ^10^, ^11^ Compared to other chondrosarcomas, primary tumours more often arise in the axial and appendicular skeleton, and at extraosseous sites such as meninges^12^, ^13^ Local recurrence and distant metastases are common and may occur up to 20 years after primary treatment. Metastatic disease at diagnosis is the main prognostic factor for inferior survival, but other factors include tumour size, axial location of the primary, and age.^3^, ^11^, ^14^ The minority of patients are cured, with 10‐year survival rates of 27–67% reported in retrospective cohorts, and even more limited in the context of metastatic disease.^5^, ^12^, ^15^ Recent SEER data demonstrates 1‐year, 5‐year, and median survival rates of 76%, 38% and 33 months respectively; with median survival of 35 months for localised disease and 18 months for metastatic disease.^3^

Previous reports describing MCS are limited to international case series and retrospective cohorts,^5^, ^10^, ^11^, ^12^, ^15^, ^16^, ^17^, ^18^ are mostly descriptive in nature, and provide limited insights into optimal treatment strategies in different clinical settings. The overall aim of this study was to determine the treatment patterns and outcomes of Australian patients treated for localised and metastatic MCS.

## 2 MATERIALS AND METHODS

We performed a retrospective study on patients of any age with MCS registered in the Australian Comprehensive Cancer Outcomes and Research Database (ACCORD); a prospective national database from six Australian sarcoma centres and by interrogation of our own institutions' pathology records between 2001 and 2022. This study was approved by the institutional ethics review board.

We collected data including baseline demographics, clinical variables including comorbidities, eastern cooperative oncology group (ECOG) performance status, stage at diagnosis, classification of bone or soft tissue sarcoma, pathology including histological diagnosis (reviewed centrally at each sarcoma centre by expert sarcoma pathologists), margin status, treatment characteristics including surgical details, radiation doses and chemotherapy regimens, best response (either by RECIST1.1, metabolic response or as documented in the clinical record) and survival status.

### 2.1 Statistical analyses

The primary outcome was overall survival (OS) defined as the time from diagnosis to death from cancer or any cause and censored at the time of last follow‐up. Secondary outcomes include progression‐free survival (PFS) defined as the time from diagnosis to recurrence (for disease localised at diagnosis), progression of disease (for those metastatic at diagnosis) or death, and, censored at the time of last follow‐up.

We used descriptive epidemiological methods to describe the cohort regarding demographics, tumour characteristics, and treatment patterns. We used the Kaplan–Meier and Cox regression methods for survival analysis and comparison between treatment subgroups using the log‐rank test (IBM SPSS Statistics, Version 28, Armonk, NY: IBM Corp., 2017).

## 3 RESULTS

We identified 22 patients diagnosed with MCS between January 2001 and March 2022 out of 5173 sarcomas from the ACCORD database and pathology review. Eight patients had an *NCOA2* gene fusion and two had NKX3.1 protein expression (Table 1). Median age at diagnosis was 28 years (range 10–59 years) and 10 patients (45%) were male. The primary site originated in the extremities in seven patients (32%), trunk in 10 patients (45%), head and neck in two patients (9%), and missing in three patients (13%). Disease was extraosseous in seven patients (32%). Further baseline characteristics are shown in Table 1. The treatment modalities and disease course are summarised in Table 2.

**TABLE 1:** Summary of baseline demographic and clinical characteristics, *N* = 22

Characteristic	No. of patients (%)
Age: Median [range], years	28 [10–59]
Male sex	10 (45)
*ECOG performance status*	
0–1	10 (45)
2–4	8 (36)
Unknown	4 (18)
*Disease stage at diagnosis*	
Localised	19 (86)
Metastatic	3 (14)
*Primary tumour type*	
Osseous	10 (45)
Extraosseous	7 (32)
Mixed	4 (18)
Unknown	1 (5)
*Primary tumour location*	
Trunk	10 (45)
Extremity	7 (32)
Head and neck	2 (9)
Unknown	3 (14)
*Size of primary tumour*	
≤8 cm	5 (23)
>8 cm	8 (36)
Unknown	9 (41)
*Site of first metastasis*	
Lung	6 (27)
Bone	4 (18)
Missing	12 (55)
*Surgical resection of primary tumour*	17 (77)
Surgical margin	
R0	5 (23)
R1	1 (5)
Unknown	11 (50)
*Chemotherapy*	13 (59)
Perioperative chemotherapy	
Yes	5 (23)
No	17 (77)
*Radiation therapy*	14 (64)
Perioperative radiation therapy	
Yes	4 (18)
No	18 (82)
*Diagnostic/molecular characteristics*	
*NCOA2* rearrangement	8 (36)
*HEY‐1*	5 (23)
*ZFP64*	1 (5)
Fusion partner not reported	2 (9)
NKX3.1 protein expression	
Positive	2 (9)
Unknown	20 (91)
Molecular panel assessed^a^	3 (15)
Unknown	16 (80)

Abbreviations: ECOG, Eastern Cooperative Group; MCS, mesenchymal chondrosarcoma; TMB, tumour mutation burden.

^a^ Case #2: CDK4/6 amplification, homozygous deletion of CDKN2A; #3 no targetable mutation, TMB 6.8; #4 COBL1‐BRAF fusion, TMB 9.

**TABLE 2:** Summary of the treatment course and outcomes of Australian patients with mesenchymal chondrosarcoma, *N* = 22

Case number	Age (years)	Sex	Tumour site at presentation	Treatment for the primary	Metastasis, recurrence (site)	Treatment for metastasis or recurrence/progression	Chemotherapy regimen	Outcome (months)
*Presented without metastasis*								
1	10	F	Vertebra	S	LR	S (×5), R (×2), CT (×1)	D	DOD (183.2)
3	25	M	Pelvis	S, R	LR + MR (bone)	S (×5), R, CT (×3)	IrT, P, VDC/IE	AWD (96.7)
5	24	M	3rd metatarsal	S				NED (26.7)
6	26	M	Maxilla	S, R, CT	LR + MR (lung)	S (×2), R	DI	DOD (211.0)
7	28	F	Maxilla	S (x2)				NED (2.7)
8	59	M	Thigh	CT, R	UK		UK	DOD (16.9)
9	18	F	Missing	CT	UK		DaDI	DOD (7.0)
10	44	F	Bone (UK)	S, R				NED (30.4)
11	30	F	Rib	S				NED (0.4)
12	20	M	Distal femur	S	MR (lung)	S (×5), R (×3), CT (×6)	M, DIV, DaDIV, VDaCE, trialx2^a^, UK	DOD (101.9)
13	33	F	Rib	S	MR (bone)	S, R (×4), CT	VDC/IE	DOD (26.1)
14	22	F	Rib	S, R				NED (21.4)
15	39	M	Vertebra	S				NED (0.0)
16	48	F	Vertebra	R, S	LR + MR (bone)	S, R		AWD (119.2)
17	14	F	Quadriceps	S				NED (59.1)
18	37	M	Buttock	S, R	LR + MR (lung)	S (×3), R (×2)		DOD (90.3)
29	38	M	Bone (UK)	CT, S	MR (bone)	CT (×2)	DzDI, trial	DOD (8.9)
21	11	M	Lower leg	S, CT			VDC/IE	NED (13.8)
22	36	F	Spine	CT, R			VDC/IE	AWD (9.4)
*Presented with metastasis*								
2	32	F	Calf (lung)	CT, S	n/a	S (×2), R (×6), CT (×4)	VIDE, VDC, trial^b^, GDo, E	DOD (34.9)
4	28	M	Pelvis (lung)	CT, R	n/a	R, CT (×4)	VIDE, VDaC, trial^b^, trial^c^, GDo	AWD (34.9)
20	27	F	Pelvis/sacrum (lung, node)	CT, R	n/a		VDC/IE	AWD (15.7)

Abbreviations: AWD, alive with disease; C, cyclophosphamide; CT, chemotherapy; D, doxorubicin; Da, dactinomycin; Do, docetaxel; DOD, dead of disease; Dz, dacarbazine; E, etoposide; G, gemcitabine; I, ifosfamide; Ir, irinotecan; LR, local recurrence; M, methotrexate; NED, no evidence of disease; R, radiation; S, surgery; UK, unknown; MR, metastatic recurrence; P, pazopanib; T, temozolamide; V, vincristine.

^a^ Investigational targeted agent.

^b^ Immunotherapy trial.

^c^ RAF inhibitor.

**FIGURE 1:**
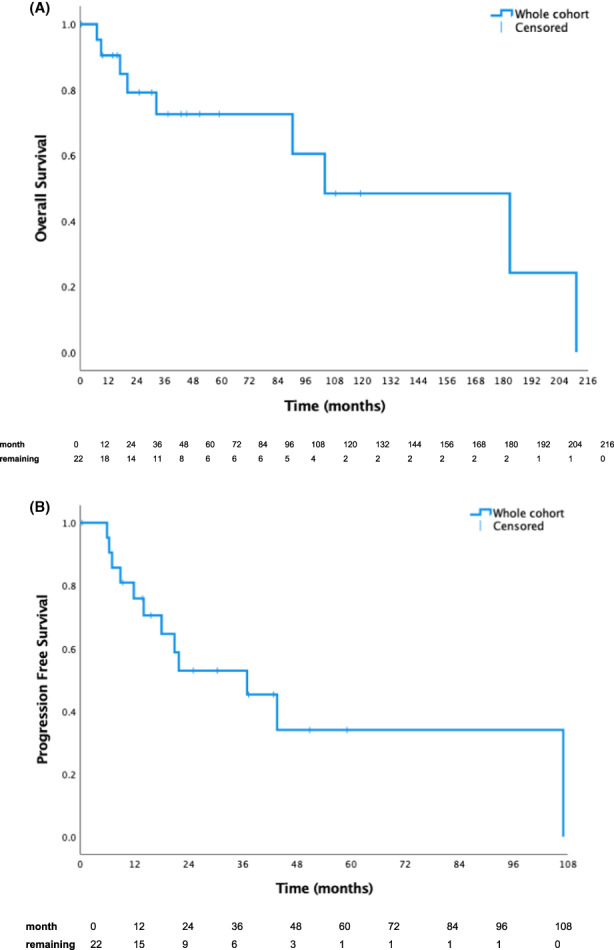
(A) This Kaplan–Meier curve illustrates the overall survival of Australian patients with mesenchymal chondrosarcoma. (B) This Kaplan–Meier curve illustrates the progression free survival of Australian patients with mesenchymal chondrosarcoma.

The median follow‐up was 50.9 months (range 0.4–210 months; Table 2). The 1‐year, 5‐year, and median OS for the whole cohort were 91%, 73%, and 104.1 months (Table 3; Figure 1A). The 1‐year, 5‐year, and median PFS were 76%, 34%, and 37.0 months (Table 3; Figure 1B).

**TABLE 3:** Summary of survival outcomes of Australian patients with mesenchymal chondrosarcoma

	Whole cohort (*N* = 22)	Disease status at diagnosis	
		Localised (*N* = 19)	Metastatic (*N* = 3)
*OS*			
Median [95%CI], m	104.1 [25.8–182.3]	182.8 [89.0–276.5]	#2 34.9 (death) #4 34.9 (alive) #20 15.7 (alive)
1‐year	91%	89%	100%
5‐year	73%	73%	50%
*PFS*			
Median [95%CI], m	37.0 [15.0–59.0]	37.0 [10.4–63.6]	#2 11.9; #4 15.2; #20 15.7
1‐year	76%	78%	67%
5‐year	34%	38%	67%

Abbreviations: CI, confidence interval; OS, overall survival; PFS, progression/disease free survival.

### 3.1 Treatment and outcomes for patients with localised disease

Disease was localised at diagnosis in 19 of 22 patients (86%). Management for localised disease (Table 2) included surgery in 16 patients (84%; surgery alone in 8), radiotherapy in eight patients (42%; one neoadjuvant and seven adjuvant) and six had chemotherapy (32%; three neoadjuvant, 2 adjuvant, 1 definitive). One patient had surgery followed by adjuvant radiation and chemotherapy. Eight patients had no evidence of recurrent disease at follow‐up.

Ten patients developed recurrence during their follow‐up, of which nine occurred beyond 2 years. Four developed both local and metastatic recurrence, three developed metastases with no local recurrence, one had local recurrence only and two had missing recurrence location.

Management for local recurrence and/or metastatic disease included surgical resection in seven patients (37%; four of five with lung metastases underwent metastatectomy), radiotherapy in seven patients (37%) and chemotherapy in five patients (26%). Four patients (21%) received all three modalities.

**FIGURE 2:**
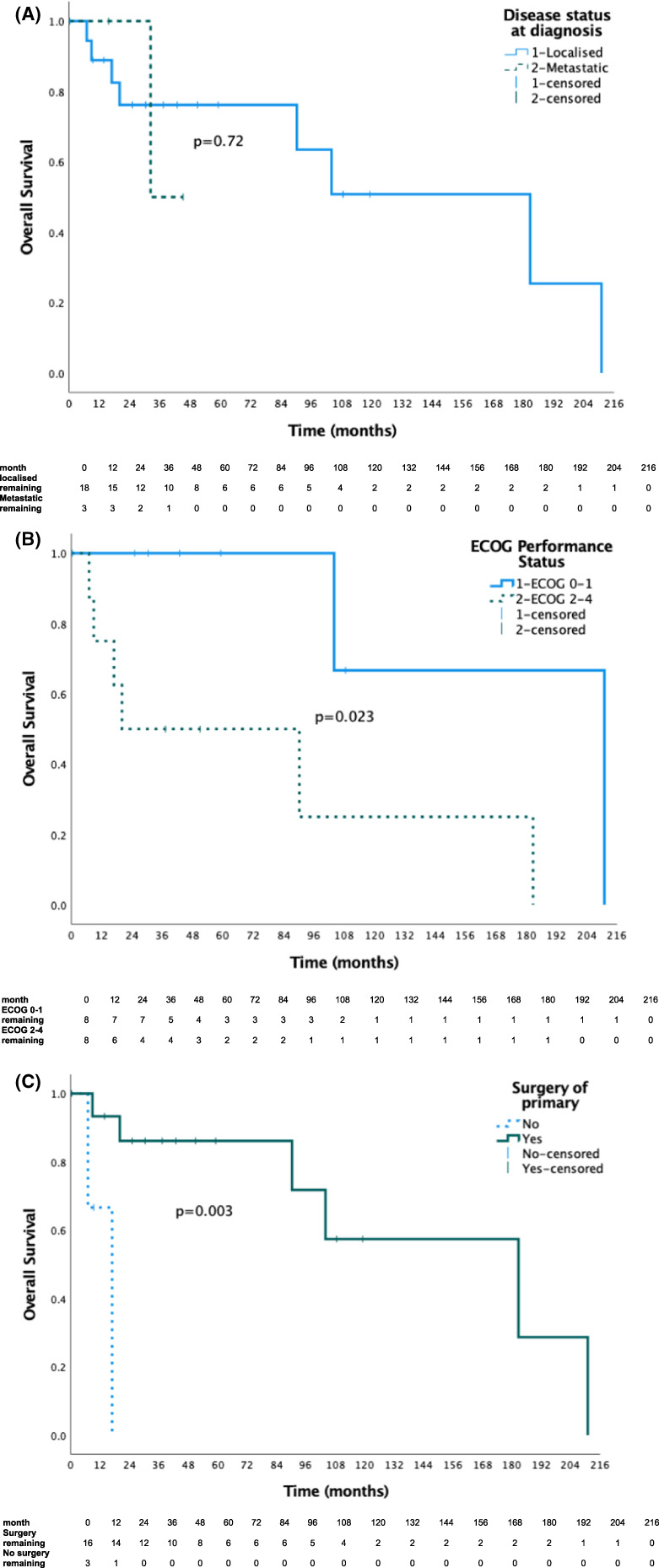
(A) This Kaplan–Meier curve illustrates the overall survival of Australian patients with mesenchymal chondrosarcoma by localised or metastatic disease status at diagnosis. (B) This Kaplan–Meier curve illustrates the overall survival in Australian patients with localised mesenchymal chondrosarcoma by ECOG group 0–1 compared to 2–3. (C) This Kaplan–Meier curve illustrates the overall survival in Australian patients with localised mesenchymal chondrosarcoma by surgical resection of the primary tumour.

Eight patients (42%) died of disease including the patient with an isolated disease recurrence. Two of the patients with both localised and metastatic recurrence remained alive at the time of writing. For patients with localised disease at diagnosis, 1‐year, 5‐year and median OS and PFS were 89%, 73%, 182.8 months and 78%, 38% and 37.0 months (Table 3; Figure 2A). Three patients who had lung metastatectomy had prolonged survival with OS 104, 109 and 211 months. Two patients who did not receive surgery to their primary had survival of 7 and 17 months.

### 3.2 Treatment and outcomes for patients with de novo metastatic disease

Three of 22 patients (14%) in our cohort were diagnosed with metastatic disease at diagnosis (Tables 2 and 3; Figure 2A). The first case was treated with a combination of chemotherapy and surgical resection of the primary tumour followed by multiple palliative surgeries, radiation, and further systemic treatments including an unsuccessful trial of immunotherapy. This patient died of disease after 34.9 months. The second and third cases remain alive at 34.9 and 15.7 months having received combination chemotherapy and radiation treatment to the primary tumour. The second and third cases have not received surgery as part of their treatment course.

### 3.3 Chemotherapy regimens used in both localised and recurrent disease settings

Thirteen of 22 patients (59%) received chemotherapy. These regimens were heterogenous but almost always contained an anthracycline (92%) including regimens typically used for Ewing sarcoma (vincristine, doxorubicin, cyclophosphamide, ifosfamide, etoposide, and irinotecan/temozolomide), soft tissue sarcoma (doxorubicin ± ifosfamide ± dactinomycin/dacarbazine/vincristine, gemcitabine/docetaxel, pazopanib), immunotherapy, and clinical trials. The chemotherapy regimens used in the primary curative setting were combination regimens with the backbone of doxorubicin and ifosfamide. These are summarised in Table 2.

Nine patients had data available for the best response to chemotherapy: Five patients had a partial response, one stable disease, one progressive disease, and two not evaluable as given adjuvant therapy.

### 3.4 Analysis of prognostic factors

Univariate analysis of prognostic factors for those with localised disease is summarised in Table 4. On univariate analysis there was a significant improvement in OS for patients with ECOG performance status of 0–1 compared to 2–3 (211 vs. 20 months; *p* = 0.039; Figure 2B) and for those who had surgical resection of the primary tumour (183 vs. 17 months; *p* = 0.003; Figure 2C). Age 28 years or less, female sex, size 8 cm or less, soft tissue/mixed tumour subtype, site of primary tumour origin in the trunk, receipt of radiation therapy suggested benefit to OS but was not significant on analysis.

**TABLE 4:** Univariate analysis of prognostic factors for overall survival in Australian patients with localised mesenchymal chondrosarcoma

	*N*	Median OS, m	HR (95% CI)	*p*‐value
Age at diagnosis				
≤28	10	183	ref	
>28	9	90	3.48 (0.61–19.74)	0.14
Sex^a^				
Female	10	183	ref	
Male	9	104	1.15 (0.26–5.22)	0.85
ECOG				
0–1	8	211	ref	
2–4	8	20	8.27 (0.97–70.44)	0.023*
Size				
≤8 cm	4	NR	ref	
>8 cm	6	20.1	77.07 (0.001–71.52x10^5^)	0.13
Tumour subtype				
Soft tissue/mixed	9	183	ref	
Bone	9	104	1.08 (0.22–5.36)	0.93
Site of origin				
Trunk	8	183	ref	
Extremity/H&N	8	104	1.57 (0.26–9.45)	0.62
Chemotherapy				
No	9	90	ref	
Yes	10	104	4.27 (0.48–38.20)	0.16
Radiation therapy				
Yes	11	183	ref	
No	8	NR	2.0 (0.28–14.36)	0.48
Surgical resection of primary				
Yes	16	183	Ref	
No	3	17	16.14 (1.40–185.47)	0.003*

Abbreviations: CI, confidence interval; ECOG, eastern cooperative group performance status; H&N, head and neck; HR, hazard ratio; NR, not reached; OS, overall survival m, months.

* *p*‐value significant if <0.05.

## 4 DISCUSSION

This is the first Australian cohort describing this ultra‐rare sarcoma and provides insight into disease presentation, patterns of care and outcomes of patients at our sarcoma centres. We identified 22 patients over 21 years with a median age of 28 years, and 86% patients had localised disease at diagnosis. Over half (59%) of the cohort experienced metastases either at diagnosis or on follow‐up. The 5‐year survival rate for this cohort was 73% with a median OS of 104.91 months (8.7 years). Most patients with localised disease were managed with wide local excision and consideration perioperative radiotherapy and chemotherapy. Patients with metastatic disease received multimodality treatment with surgery, radiotherapy, and predominantly anthracycline‐based chemotherapy regimens. We found improved OS in patients with good performance status and those who had surgical resection of the primary.

MCS exhibits more aggressive behaviour and worse survival outcomes compared to conventional chondrosarcoma.^3^, ^10^ Patients are younger and a higher proportion of tumours are extraosseous.^5^, ^10^ Additionally, MCS is considered to be more chemo‐ and radiosensitive.^10^, ^12^, ^15^, ^16^ Chemotherapy regimens typically used are as those for Ewing sarcoma.^11^, ^12^, ^19^ There are also reports of responses to novel agents such as Trabectedin,^8^, ^20^ and targeted agents including immunotherapeutics.^21^, ^22^

The literature in this area is summarised in an additional file along with results from our study for comparison (Supporting Information S1).^2^, ^3^, ^5^, ^10^, ^11^, ^12^, ^14^, ^15^, ^16^, ^17^, ^18^, ^23^, ^24^, ^25^, ^26^, ^27^, ^28^, ^29^, ^30^, ^31^

Most patients had localised disease at diagnosis, and consistent with the literature about a third developed local recurrence and just over half developed metastatic disease at follow‐up, suggesting the presence of occult metastases. Most relapses occurred beyond 2 years after initial management with curative intent, suggesting maintenance of regular imaging is important to detect later relapses. Even patients with curable local disease have an aggressive disease course with almost half dying of disease. Most cases that underwent lung metastatectomy in the relapsed setting had prolonged survival for more than 5 years, which supports the role of definitive treatment for oligometastatic disease.^32^, ^33^, ^34^

Performance status is a well‐established prognostic factor in advanced cancer and we expected improved OS for those with better ECOG as demonstrated in our cohort.^35^ This confirms the selection bias inherent in retrospective data that patients with improved fitness and more favourable disease biology will live longer and thereby receive more treatments. The patients who did not have surgery in the curative setting were likely not fit and therefore, had poorer survival.

The literature is not consistent in outcomes based on patients' age. Some studies have suggested a poorer outcome in those under 30 years.^25^ Our data suggested improved OS in the younger cohort, although underpowered, which has also been shown in the literature but when high dose multiagent chemotherapy including autologous stem cell transplant has been used.^12^

In our cohort, patients with tumours originating from the trunk have longer survival than those originating from the extremity or head and neck. This is in contrast to other published literature where axial tumours are expected to have a worse prognosis as surgical resection is more challenging compared to extremity, head and neck tumours.^23^, ^27^

Size is another well‐established prognostic factor for sarcomas and forms the basis for staging.^36^, ^37^ Our study showed similar outcomes between patients with tumours 8 cm or less compared to those more than 8 cm, which is in contrast to the literature.^14^, ^23^ Our result is likely due to the small sample size but raises the consideration that early occult metastatic spread could contribute to poor prognosis regardless of primary tumour size.

Surgical resection is well established as the mainstay of curative treatment for MCS which is confirmed by our results. However, given the high relapse rate, there is a question of whether additional local therapies can help improve the durability of disease control. This is important in cases where wide margins are not feasible.^10^ Adjuvant radiation therapy is typically reserved for close margins and contaminated local spaces to improve locoregional control^38^, ^39^ or in the advanced setting for palliation symptoms such as pain or bleeding. Harwood and Kawaguchi both advocated for the role of adjuvant radiation to prevent local recurrence.^25^, ^27^

We agree with the proposal in the literature that perioperative chemotherapy in the localised setting may reduce recurrence and improve cure rates given a high rate of metastatic relapse which may relate to inadequately treated micrometastatic disease.^10^, ^27^ Studies evaluating perioperative chemotherapy in similar disease groups such as Ewing sarcoma have demonstrated survival advantages.^39^, ^40^

Chemotherapy agents used in the first line setting were heterogeneous. The literature is not clear which of these agents is most active, although most of our patients received an anthracycline which is the backbone of most sarcoma regimens.^10^ Multiple agents over single‐agent treatment may have benefits for chondrosarcoma including MCS.^17^, ^18^, ^25^ Data for targeted agents is very limited, but there is interest in the role of pazopanib in conventional chondrosarcoma,^22^, ^36^, ^41^ and a biologic rationale for multikinase inhibition in MCS.^42^ The role of immunotherapy in MCS remains to be defined.^21^

A limitation to this study is its retrospective nature which is prone to missing and incomplete data. Diagnostic data such as gene fusion status was missing in some of our patients due to the lack of commercial availability of gene probes at the time of diagnosis. We have used a more recent diagnostic immunostain of NKX3.1 for clarification for some of these cases and emphasise that cases extracted from the ACCORD database are diagnosed at centralised services with expert pathologists who are able to confidently diagnose this disease without additional testing for classic cases.

We have limited our univariate analysis to localised patients only, however, the three metastatic cases provide important information on sequencing of treatments and including surgical resection of the primary where suitable. We have not performed multivariate analysis due to low sample size and model instability.

The novelty of our cohort is that it is mostly localised at diagnosis and highlights the high metastasis rate which likely reflects underlying occult metastatic disease. This provides rationale for earlier multiagent chemotherapy using regimens with documented activity in the metastatic setting.

## 5 CONCLUSIONS

Further prospective study is needed to delineate the optimal sequence of therapies. Given the rarity of MCS, prospective controlled studies are unlikely to be feasible. This is a challenge for researchers of ultra‐rare diseases. International collaboration is often required and early phase basket trials and regulatory approaches that give patients with advanced disease access to novel therapies is one strategy.^4^ It is also imperative that each individual patient's case is captured by databases such as ours with support for periodic interrogation and knowledge transfer.

This study is the first report of contemporary Australian treatment patterns of MCS and confirms an aggressive disease entity in a young population. This justifies an aggressive therapeutic approach utilising all treatment modalities. Understanding treatment patterns and outcomes helps promote clinical decision‐making and the design of trials for novel therapeutic strategies.

## AUTHOR CONTRIBUTIONS

**Madeleine C Strach:** Conceptualisation, methodology, investigation, data curation, formal analysis, writing‐original draft, writing – review and editing. **Peter S Grimison:** Conceptualisation, methodology, investigation, data curation, writing – review and editing, supervision. **Angela Hong:** Investigation, data curation, writing – review and editing. **Richard Boyle:** Investigation, data curation, writing – review and editing. **Paul Stalley:** Investigation, data custodian, writing – review and editing. **Rooshdiya Karim:** Investigation, data curation, writing – review and editing. **Elizabeth Connelly:** Investigation, data curation, writing – review and editing. **Susie Bae:** Data custodian, data curation, writing – review and editing, supervision, project administration. **Jayesh Desai:** Data custodian, data curation, writing – review and editing. **Philip Crowe:** Data custodian, data curation, writing – review and editing. **Nimit Singhal:** Data custodian, data curation, writing – review and editing. **Vivek A Bhadri:** Conceptualisation, methodology, investigation, data curation, writing – review and editing, supervision.

## CONFLICT OF INTEREST

Madeleine C Strach has served on the advisory board for Specialised Therapeutics and received personal fees from Specialised Therapeutics. Angela Hong has received a consultancy fee from Bayer and Provectus. The other authors made no disclosures.

## TWEET

Outcomes from an Australian study on ultra‐rare mesenchymal chondrosarcoma confirm poor survival. Improved treatments and global collaboration are needed to improve patient outcomes.

## Supporting information


**Appendix S1** This table summarises our literature review of studies evaluating clinical outcomes of patients with mesenchymal chondrosarcoma. This additional file is a summary of the literature published to date evaluating treatments and outcomes in patients with mesenchymal chondrosarcoma.

## Data Availability

The data that support the findings of this study are available from the corresponding author upon reasonable request.
