# Supplementary material for: Mesenchymal chondrosarcoma: An Australian multi‐centre cohort study
Source: Cancer Med. 2022 May 23;12(1):368–78. doi: 10.1002/cam4.4849 (PMC9844591; doi:10.1002/cam4.4849)
Supplement: Supplementary file 1 — Appendix S1 This table summarises our literature review of studies evaluating clinical outcomes of patients with mesenchymal chondrosarcoma. This additional file is a summary of the literature published to date evaluating treatments and outcomes in patients with mesenchymal chondrosarcoma. [file CAM4-12-368-s001.pdf]

Supporting information 1. Summary of literature review of studies evaluating treatments and clinical outcomes of patients with mesenchymal chondrosarcoma.

Author, Year	Study design	Population (Male)	Median age (range), y	Metastatic at diagnosis (site)	Treatment	Chemotherapy agents	Median OS, m	Median PFS, m	Comment
Strach et al, (this study)	Retrospective review 2001-2022	22 MCS (45%)	28 (10-59)	3 (Lung, node)	17 S<br>2 preop CT<br>8 palliative CT<br>13 RT	D<br>DI<br>+Dz<br>+Vinc<br>+Da<br>VDC/IE<br>VIDE<br>IrT<br>M<br>VDaC<br>E<br>GDo<br>P<br>Durvalumab + Tremilimumab<br>Investigational	104	34	
Van Maldegem et al, 2019[16]	Retrospective review of 4 sarcoma centres 1980-2016	25 unresectable (36%)	27.5 (7-48)	NR	25 multidrug CT	D<br>+Cis<br>++M<br>+C<br>I<br>CisDzV<br>Anastrazole<br>Anti-TIM3<br>IDH inhibitor<br>Durvalumab + Mogamulizumab		Mean 6.7	
Amer et al, 2019[3]	Retrospective review of SEER database 1973-2013	226 MCS (54%)	32 (NR)	24	86 RT	Not reported	34		CT and S not reported
Tsuda et al, 2017[22]	Retrospective review, 1980-2013	57 MCS	33 (6-73)	17	Localised<br>34 S<br>13 CT+S<br>10 S+RT	D<br>I<br>Cis<br>carboplatin	5y 66%<br>10y 56%	5y MFS 26%	

Supporting information 1. Summary of literature review of studies evaluating treatments and clinical outcomes of patients with mesenchymal chondrosarcoma.

Author, Year	Study design	Population (Male)	Median age (range), y	Metastatic at diagnosis (site)	Treatment	Chemotherapy agents	Median OS, m	Median PFS, m	Comment
					6 RT<br><br>Advanced<br>16 CT	E<br>C<br>V<br>M<br>Da	CT 91%<br>No chemo 57%		
Morioka et al, 2016[23]	Randomised phase 2 prospective study of TRS 2012-2014	5 trabectedin (3 MCS)<br>3 BSC (3 MCS)	<40 (MCS)	all	MCS:<br>4/6 S<br>6 CT (3 preop)<br>1 RT (only reported in case study)	D<br>DCis<br>VDC<br>VDC/IE	Trabectedin: 26<br>BSC: not reported	Trabectedin: 13<br>BSC: 1 (p)	1 PR, 2 SD<br><br>Analysis of trabectedin grouped MCS with EMCS
Schniederman et al, 2016[13]	Retrospective review of SEER database, 1973-2011	205 (114)	37	Not reported	Not reported	Not reported	64m<br><br>5y 51%<br>10y 43%		Multivariate analysis on 104 due to missing data (tumour size)
Xu et al, 2015[9]	Systematic review 1994-2014 (included case reports)	107 MCS from 18 articles (56%)	88 <50yrs	24	93% S<br>63% CT<br>38% RT<br>40% S+CT<br>7% S+RT<br>15% S+CT+RT		80<br><br>5y OS 55%<br>10y OS 44%<br><br>(no differance OS local v met p=0.095)	Median EFS 58	Excluded cases/series if patients died in 3m of FU
Frezza et al, 2015[10]	Retrospective review of EMSOS cases 1971-2012	113 (60)	30 (11-80)	17 (lung, bone)	Localised (96)<br>95 S<br>54 CT (21 preop, 30	Localised 96% anthracycline<br>70% alkylating	17 years<br>Localised 20y<br>Metastatic 3y	Localised 7yrs	Likely duplication of patients from previous analysis, 128

Supporting information 1. Summary of literature review of studies evaluating treatments and clinical outcomes of patients with mesenchymal chondrosarcoma.

**Author, Year**	**Study design**	**Population (Male)**	**Median age (range), y**	**Metastatic at diagnosis (site)**	**Treatment**	**Chemotherapy agents**	**Median OS, m**	**Median PFS, m**	**Comment**
					postop, 3 pre and postop)<br>40 RT (38 postop, 1 pre and postop)<br><br>Metastatic (17)<br>2 S (primary, metastatectomy) postop RT<br>3 S (primary)<br>1 RT (primary)	Metastatic:<br>Trabectedin<br>EpI			cases, 15 excluded as no information
Kawaguchi et al, 2014[24]	Retrospective review 1979-2010	37 (20)	33 (11 - 65)	7 (bone, breast, colon)	Primary:<br>8 S<br>13 S+CT (10 preop and 3 postop)<br>5 S+RT<br>8 S+CT+RT (1 preop CT+ RT, 2 preop CT and postop RT, 1 preop RT and postop CT, 4 postop CT+RT)<br>1 CT<br>1 RT<br>1 BSC	D (21)<br>+C+Bleomycin<br>+Cis<br>+CDz<br>DI<br>+V<br>+Dz<br>Cis<br>+Do<br>+DzM	5y 51%<br>10y 37%	DFS<br>5y 23%<br>10y 5%	Localised:<br>RT improved local RFS (p=0.037)<br><br>No influence of CT on survival (p0.397)
Shakked et al, 2012[25]	Retrospective review of records of senior bone pathologist 1964-2009	20 (9)	24 (7-37)		9 S<br>3 S+CT<br>1 S+RT<br>4 S+CT+RT	VDC/IE<br>IE<br>DI	Not reported for entire cohort		

Supporting information 1. Summary of literature review of studies evaluating treatments and clinical outcomes of patients with mesenchymal chondrosarcoma.

Author, Year	Study design	Population (Male)	Median age (range), y	Metastatic at diagnosis (site)	Treatment	Chemotherapy agents	Median OS, m	Median PFS, m	Comment
Italiano et al, 2013[17]	Retrospective review of 15 centres 1988-2011	112 CS – 17 MCS<br>(CS: 62%)	CS: 52 (17-84)	All (135 lung, 40 bone, 8 liver, 39 other)	CT:<br>Multidrug – 98<br>Single – 82<br>S: not stated	D<br>+I<br>+CisI<br>+Cis<br>+other<br>PegD<br>I<br>+Cis<br>GDo<br>Imatinib<br>Sirolimus+C<br>Trabectedin<br>Dz<br>E<br>IrT			Palliative surgery prognostic for OS<br>(not hedgehog study)
Dantonello et al, 2008[11]	Retrospective review of prospective CWS/COSS database 1977 - 2006	15 (33%)	16.6 (1-25)	1	S: All (8 complete)<br>CT: 13<br>RT: 6	D<br>+DaCV<br>+DaIV<br>+IMCis<br>+IMCis, carboE→autosct<br>Da<br>+<br>carboEIV→auto SCT<br>+DEIMV	10y OS 67%	10y EFS 53%	7 induction CT (4 >50% tumour volume regression or histologic devitalisation)
Cesari et al, 2007[15]	Retrospective database review 1959-2003	28 (39%)	31 (20-71)	6 (Bone, lung, kidney, spleen, pancreas)	S: 24<br>RT: 5<br>CT: 12 (9 localised, 3 metastatic)	SE-91 protocol<br>DI<br>+CisM	10y OS<br>Chemo 31%<br>No chemo 19%<br>(p=0.008)		

Supporting information 1. Summary of literature review of studies evaluating treatments and clinical outcomes of patients with mesenchymal chondrosarcoma.

Author, Year	Study design	Population (Male)	Median age (range), y	Metastatic at diagnosis (site)	Treatment	Chemotherapy agents	Median OS, m	Median PFS, m	Comment
Nakashima et al, 1986[14]	Case series retrospective review	111 (47%)	[a](5-74)	3 (2 lung, 1 LN)	[b]10 S+RT+CT<br>19 S+CT<br>8 S+RT		29% by 17y	DFS 20% (by 17y)	
Huvos et al, 1983[5]	Retrospective review 1931-1981	35 (57%)	mean 25 (6-70)	5 (2 bones, 1 lung, 1 chest wall, 1 breasts)	4 CT+S<br>8 RT+S<br>1 S+CT<br>10 S+RT	M	5yr OS 42% (n=32, 3 treatment to recent)		
Misc authors 1959-1983[2, 26-30]		127	17-30						Not included here as historical
**TOTAL**		**906**	**30 (1-84)**				**10y 19-67%**		

Abbreviations: MCS, mesenchymal chondrosarcoma; S, surgery; RT, radiation therapy; CT, Chemotherapy; preop, preoperative; postop, postoperative; DFS, disease free survival; PFS, progression free survival; OS, overall survival; MFS, metastasis free survival; LN, lymph node; TRS, translocation-related sarcomas; BSC, best supportive care; ; D, doxorubicin; Ir, irinotecan; T, temozolamide; P= pazopanib; V, vincristine; Da, dactinomycin; I, ifosfamide; E, etoposide; Dz, dacarbazine; M, methotrexate; C, cyclophosphamide; G, gemcitabine; Do, docetaxel; Cis, Cisplatin; Ep, Epirubicin; PegD, pegylated doxorubicin; AutoSCT, autologous stem cell transplant; SE-91 protocol[42]

[a]average age not provided

[b]treatment and follow up available for 78 patients
